# SIME: synthetic insight-based macrolide enumerator to generate the V1B library of 1 billion macrolides

**DOI:** 10.1186/s13321-020-00427-6

**Published:** 2020-04-10

**Authors:** Phyo Phyo Kyaw Zin, Gavin Williams, Denis Fourches

**Affiliations:** 1 Department of Chemistry, North Carolina State University, Raleigh, NC USA; 2 Bioinformatics Research Center, North Carolina State University, Raleigh, NC USA; 3 Comparative Medicine Institute, North Carolina State University, Raleigh, NC USA

**Keywords:** Macrolides, PKS enumerator, In silico chemical library software, Polyketides

## Abstract

We report on a new cheminformatics enumeration technology—SIME, synthetic insight-based macrolide enumerator—a new and improved software technology. SIME can enumerate fully assembled macrolides with synthetic feasibility by utilizing the constitutional and structural knowledge extracted from biosynthetic aspects of macrolides. Taken into account by the software are key information such as positions in macrolide structures at which chemical components can be inserted, and the types of structural motifs and sugars of interest that can be synthesized and incorporated at those positions. Additionally, we report on the chemical distribution analysis of the newly SIME-generated V1B (virtual 1 billion) library of macrolides. Those compounds were built based on the core of the Erythromycin structure, 13 structural motifs and a library of sugars derived from eighteen bioactive macrolides. This new enumeration technology can be coupled with cheminformatics approaches such as QSAR modeling and molecular docking to aid in drug discovery for rational designing of next generation macrolide therapeutics with desirable pharmacokinetic properties.
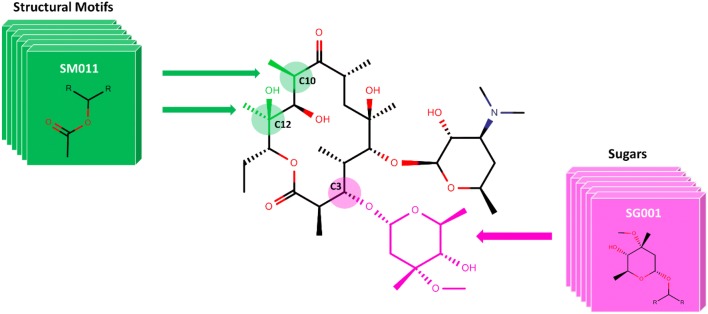

## Introduction

Macrolides are at least 12-membered glycosylated macrolactone rings [1] wherein 12- to 16-membered macrolides are widely known as a family of antibiotics [2, 3] (e.g. erythromycin, pikromycin, clarithromycin, azithromycin). Macrolides belong to a large family of protein synthesis inhibitors and other biologically activity members known as polyketides: structurally diverse and complex natural products with important therapeutic properties [4]. They display diverse biological properties such as antibiotic, antifungal, antimicrobial, anticancer, and immunosuppressant activity [2]; they thus have gained a critical interest in the pharmaceutical industry to be pursued as starting points for the development of novel antibacterial agents [4]. The advancements in combinatorial biosynthesis have made it possible to genetically engineer biosynthetic pathways to natural products to produce unnatural/modified macrolides based on a molecular scaffold provided [5].

Macrocyclic structures are known for their ability to bind to large, featureless, almost undruggable protein binding pockets [6], and their unusual physicochemical properties commonly far exceed all of the thresholds, structural alerts, and other empirical rules for estimating druglikeness (such as Lipinski [7] and Veber [8]). In fact, the unique nature of the ring scaffold makes them highly desirable due to the resulting high structural pre-organization and rigid conformations [9, 10]. The rigidity of the macrocyclic backbone limits the flexibility of certain structural motifs, thereby reducing undesirable off-target interactions and the associated entropic costs, which sequentially increases binding affinity [6]. Macrocycles also possess “*chameleonic*” properties that enable conformational flips driven by intra-hydrogen bonding and polarity of the solvent environment, yielding a significant impact on bioavailability and permeability [11]. Additionally, they are known for their stability to proteolytic degradation in intricate biological surroundings, higher membrane permeability and metabolic stability [9], which are important druglike properties. The mechanistic insights and molecular properties that allow for such favorable bioavailability and pharmaceutical properties are not yet fully understood, which hinders the innovation and exploitation of this structural class for novel polyketide antibiotics [10, 12, 13]. Hence, studying macrolides has potential to yield important findings that could help identify vital key characteristics for novel drug designs and development. However, they are still under-exploited in part due to limited tools and databases to investigate structural features and relationships of macrolides. In fact, there has been little progress in the development of newer macrolide drugs up until very recently [14]; even though the first macrolide, erythromycin, was discovered in 1952 [15].

The search for novel macrolide therapeutics can be facilitated by cheminformatics methods such as global/local structural enumeration, and virtually screened chemical libraries which can be modeled against biological targets of interest. Protein–ligand complex binding interactions and structure–activity relationships (SAR) can be extracted from such studies and used to optimize lead compounds with enhanced inhibition potencies and binding affinities. However, the actual lack of in silico macrolide libraries in public repositories has been a major challenge in researching this unique structural class.

That was the rationale for our recent study in which we introduced a cheminformatics approach based on the concept of macrolide structural motif (SM; building blocks) to efficiently generate large in silico, screening-ready libraries of macrolides with complex user-defined structural constraints [16]. We also reported on the resulting screening-ready chemical database V1 M containing 1 million macrolide scaffolds with SMs extracted from eighteen experimentally confirmed macrolides. Two major weaknesses regarding the first approach were related to the exclusion of sugars (even though they have been shown to be critical for the biological activity [17]) and the actual synthetic feasibility based on biosynthetic engineering approach. The V1 M macrolide scaffolds did not contain sugars, a significant component sometimes contributing to one or two-thirds of the binding energy [17, 18]. In addition, all the SMs were added one after another; hence, it might result in the arrangement of chemical components that were unstable or couldn’t be synthesized via biosynthetic engineering techniques. However, a virtual screening library can only be truly useful if the hit compounds discovered during the process can de facto be synthesized and experimentally tested.

Herein, we propose a more advanced, cheminformatics enumeration approach with enhanced biosynthetic feasibility and full integration of various SMs as well as sugars of interest to scaffolds. This approach is inspired directly by the actual enzymatic assembly machinery of polyketides. The insights from the biosynthetic engineering studies of macrolide synthesis can be applied to our SIME (synthetic insight-based macrolide enumerator) technology to construct in silico chemical libraries that can in principle be experimentally synthesized. In this study, we also present the resulting V1B chemical library, the largest freely available virtual database of macrolides to date. V1B contains 1 billion fully assembled macrolides with their attached sugars, constructed based on the scaffold of Erythromycin. We studied the distribution of important chemical properties known to affect drug efficiency, absorption and bioavailability such as molecular weight—MW, polar surface area—PSA, hydrophobicity—SlogP, hydrogen bond acceptors—HBA, hydrogen bond donors—HBD, and rotatable bonds—NRB. The distribution of the aforementioned properties of V1B were compared to MacrolactoneDB, a database of existing macrolactones mined from public repositories from another study.

Overall, V1B was generated as a *proof*-*of*-*concept* study (1) to help illustrate the features of SIME, (2) to encourage computational and experimental synthetic scientists to custom design virtual chemical libraries of macrolide scaffolds suited for their project needs, and (3) to further our understanding of macrolides for the pharmaceutical advancement and search of novel therapeutics. The V1B sample library is freely available in the Supplementary Material of this manuscript (Additional file 1). Moreover, SIME (and the full V1B library) is also freely available for download (http://www.fourches-laboratory.com/software and https://github.com/zinph/SIME). We believe this new virtual library of publicly available macrolide scaffolds will enable and inspire other molecular modeling studies.

## Results

### Parameter settings for V1B

**Fig. 1:**
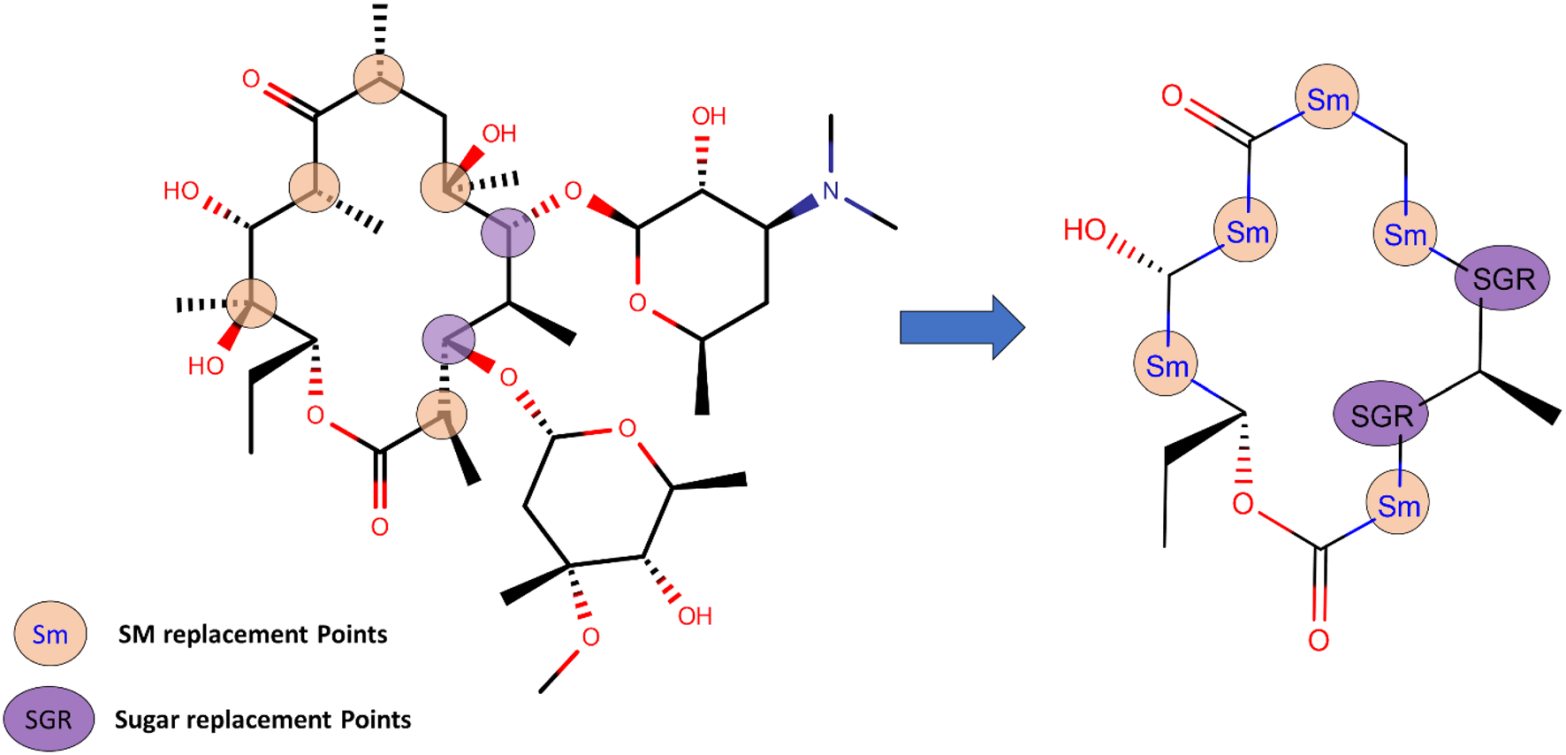
Example structure of erythromycin core with possible structural motif and sugar replacement positions

**Fig. 2:**
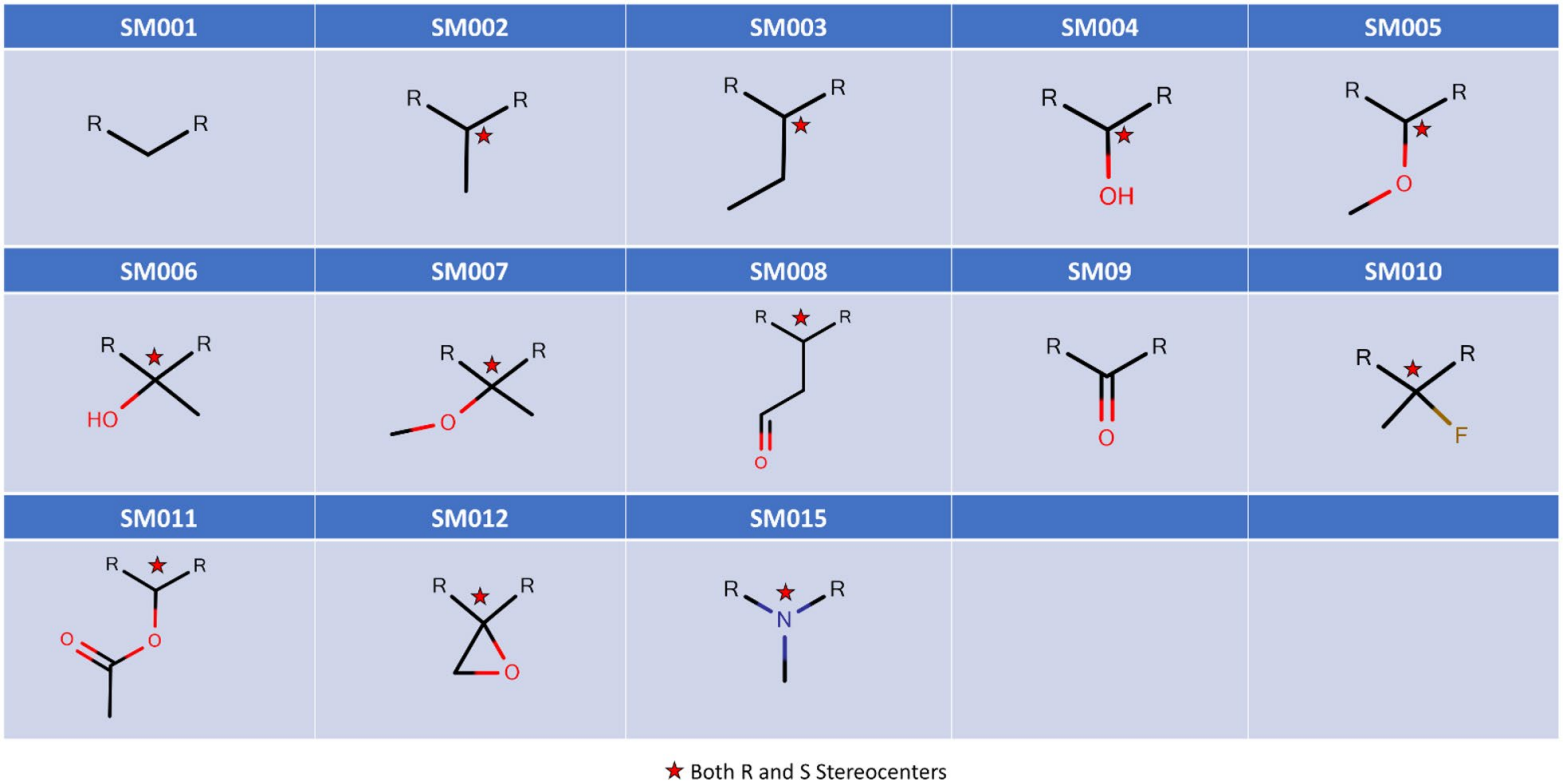
Structures of 13 SMs used to generate V1B Library

**Fig. 3:**
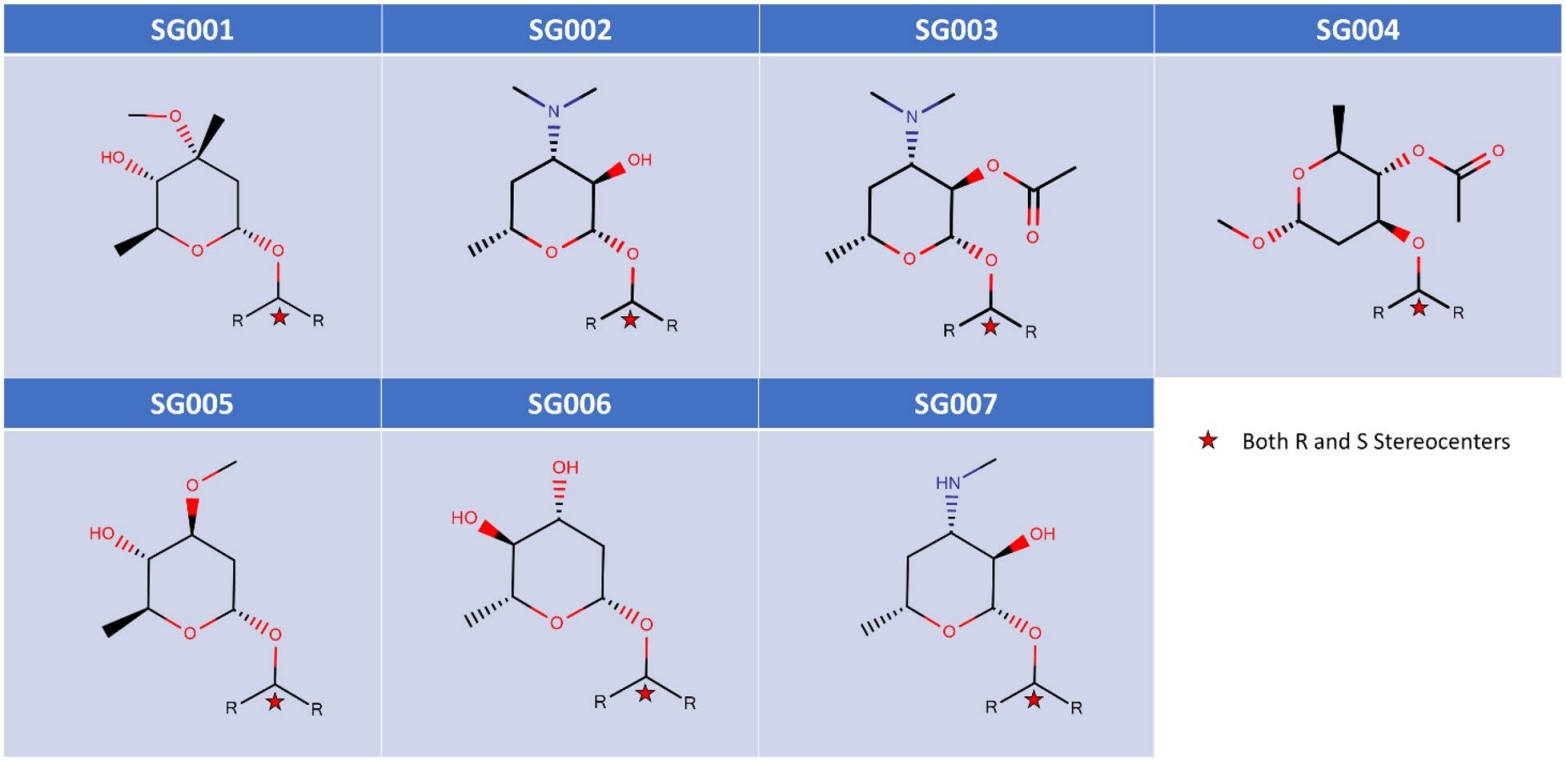
Structures of seven sugars used to generate the V1B Library

The structural core of Erythromycin with five possible SM and two sugar substitution points **(**Fig. 1) was used as the major scaffold template for generating V1B. The entire V1B database containing 1 billion compounds is freely available via GitHub (https://github.com/zinph/SIME). In our previous research, we compiled and studied eighteen experimentally confirmed bioactive macrolides (BMs) from different studies [1, 18–25] and extracted nine “common” and seven “rare” SMs from the scaffolds of 18 BMs (structures shown in Fig. 4 of [16]). The distribution analysis of SMs found in 18 BMs can be found in the cheminformatics study by Zin et al. [16]. Among the aforementioned SMs, we selected 13 SMs; nine “common” SMs and four “rare” SMs (Fig. 2), as inputs for SMs of interest in SIME. We additionally extracted seven sugars from the same 18 BMs to incorporate into V1B macrolide scaffolds (Fig. 3).

To recapitulate, 13 SMs (Fig. 2) and seven sugars (Fig. 3) extracted directly from 18 BMs from the cheminformatics study by Zin et al. [16] were used to substitute and enumerate the novel macrolide structures in V1B. The option to enumerate all possible stereocenters at the connecting points to the macrolide core was enabled. The number of maximal repeats for the same SMs per scaffold was set to three, the minimal number of sugars per scaffold was set to one. The library size was restricted to 1 billion molecules.

Since there are eleven out of 13 possible stereocenters at the connecting points for SMs and seven out of seven for sugars, there are in fact 24 SMs (2 × 11 SMs with possible R, S configurations + 2 SMs without R,S configurations) and 14 sugars (2 × 7 with possible R, S configurations). Based on this, the possible chemical space of macrolides based on these given input parameters would contain 1.57 billion compounds; 24 total SMs at five substitution points with 3 repeatable SMs (24^3^ × 23 × 22) × 14 total sugars in at least one of two substitution points (14 × 1 + 1 × 14 + 14 × 14). Since V1B contained 1 billion compounds, it covered 63.8% ($\frac{{10^{9} }}{1,566,867,456}$) of that possible chemical space.

### Distribution of V1B molecular properties

**Fig. 4:**
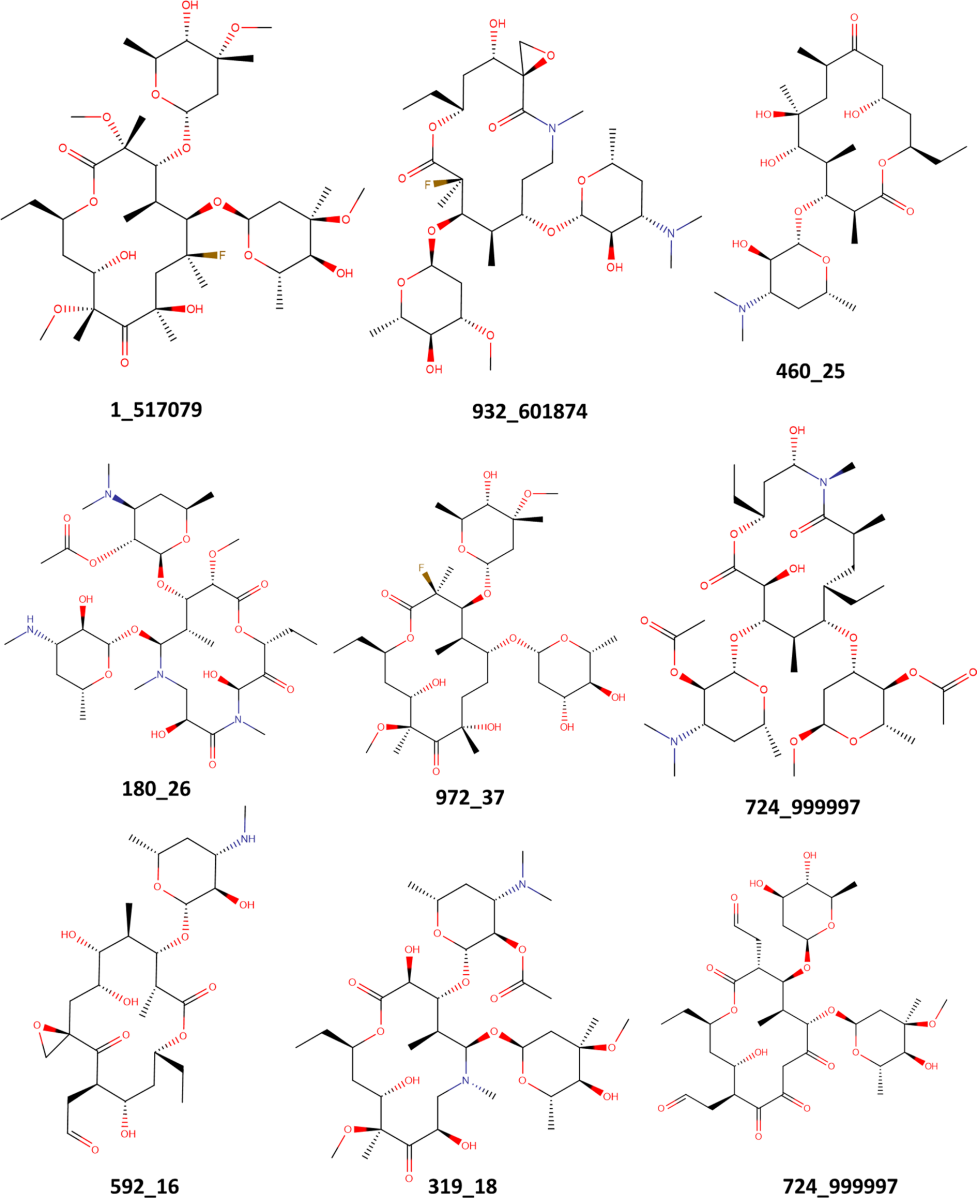
Randomly picked example macrolide structures from V1B. The first set of digits correspond to the name of the files and the second set the row ID of the compound

To save time and computational power in conducting the distribution analysis of the entire V1B library, we applied a stratified random sampling to V1B and extracted 1 million representative macrolides (V1B sample—freely available in the Supplementary Material of this paper). V1B was exported as 1000 output files, each of them contained 1M systematically enumerated macrolides. From each file, we randomly sampled 1000 macrolide structures, totaling a final set of 1M macrolides for distribution analysis. These 1 million macrolides should reasonably represent the macrolide structures from the entire V1B library. Nine macrolide structures randomly sampled from V1B are shown in Fig. 4.

**Fig. 5:**
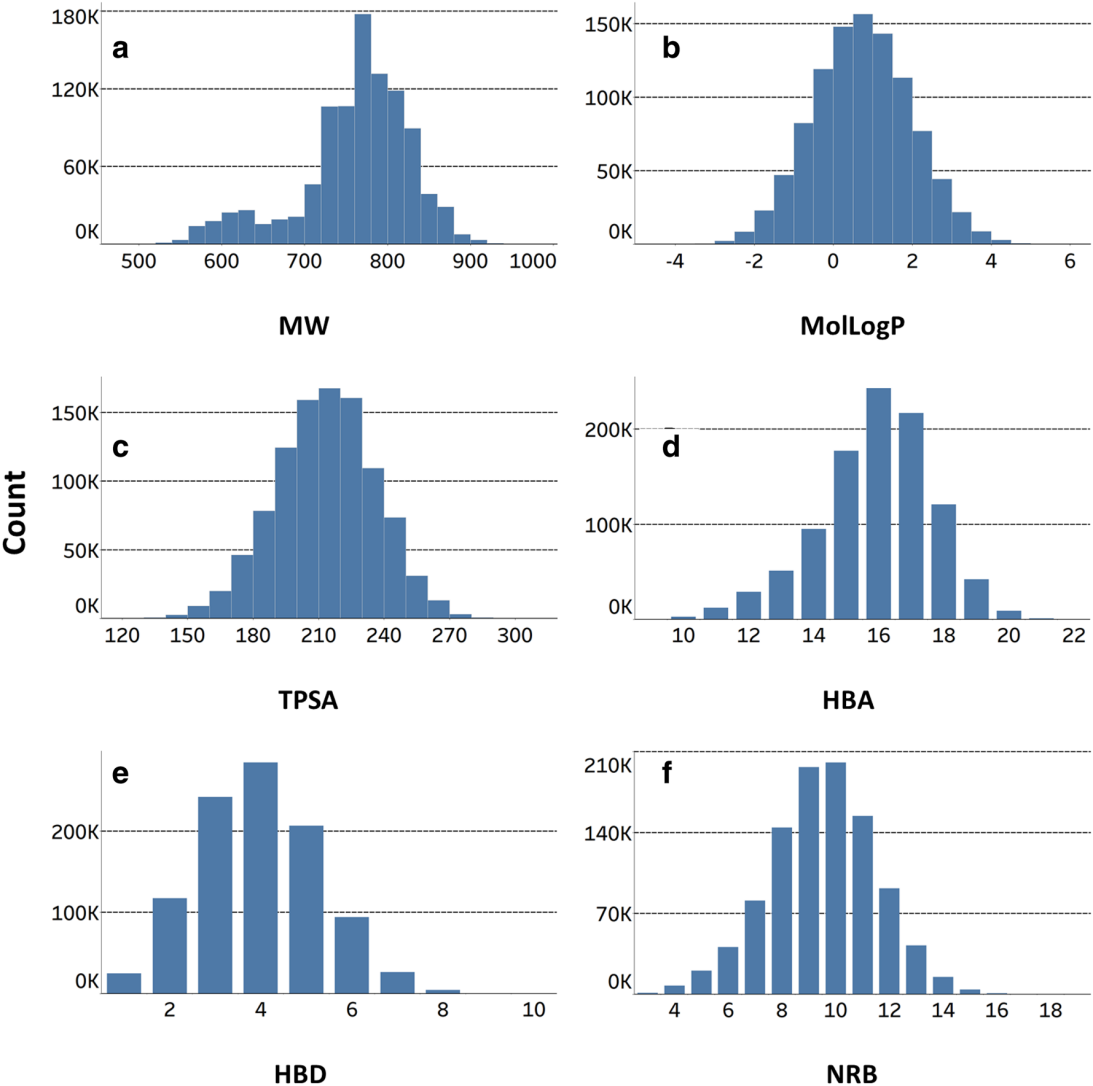
Distribution of molecular properties in V1B: **a** MW—molecular weight, **b** MolLogP—calculated water/octanol partition coefficient, **c** TPSA—topological polar surface area, **d** HBA—hydrogen bond acceptors, **e** HBD—hydrogen bond donors, **f** NRB—rotatable bonds

As in the analysis of V1M library conducted by Zin et al. [16], we studied the distribution of six important molecular properties for the V1B sample: molecular weight—MW, hydrophobicity—MolLogP, topological polar surface area—TPSA, hydrogen bond acceptors—HBA, hydrogen bond donors—HBD and rotatable bonds—NRB (Fig. 5). More descriptive statistics on the aforementioned properties are provided in Additional file 2: Table S1. We also compared them to Lipinski and Veber’s rules of drug likeness and bioavailability even though most bioactive macrolides with reasonable bioavailability disobey Lipinski’s rules (e.g., macrolides with MW > 500) [9].

MW followed a slightly bimodal distribution and ranged from 488.62 to 975.09 g mol^−1^ with a mean of 761.25 ± 68.17 g mol^−1^ (Fig. 5a, Additional file 2: Table S1). 100% exceeded Lipinski’s MW of 500. MolLogP followed a gaussian distribution with a range from − 4.30 to 5.66, and a mean of 0.73 ± 1.24 (Fig. 5b, Additional file 2: Table S1). Interestingly, less than 1% exceeded SlogP of 5. TPSA followed a gaussian distribution ranging from 125.76 to 306.87 Å^2^ with a mean of 213.09 ± 22.88 Å^2^ (Fig. 5c, Additional file 2: Table S1). 99.92% exceeded Lipinski’s TPSA of 140. HBA followed a slightly left-skewed distribution and ranged from 9 to 22 with a mean of 15.91 ± 1.77 (Fig. 5d, Additional file 2: Table S1). An overwhelming 99.7% violated HBA of at most 10. HBD followed a gaussian distribution with a range from 1 to 10 and a mean of 3.94 ± 1.35 (Fig. 5e, Additional file 2: Table S1). 12.53% disobeyed HBD of at least 5. NRB followed a gaussian distribution and ranged from 3 to 19 with a mean of 9.50 ± 2.00 (Fig. 5e, Additional file 2: Table S1). 30.82% surpassed NRB of 10.

**Fig. 6:**
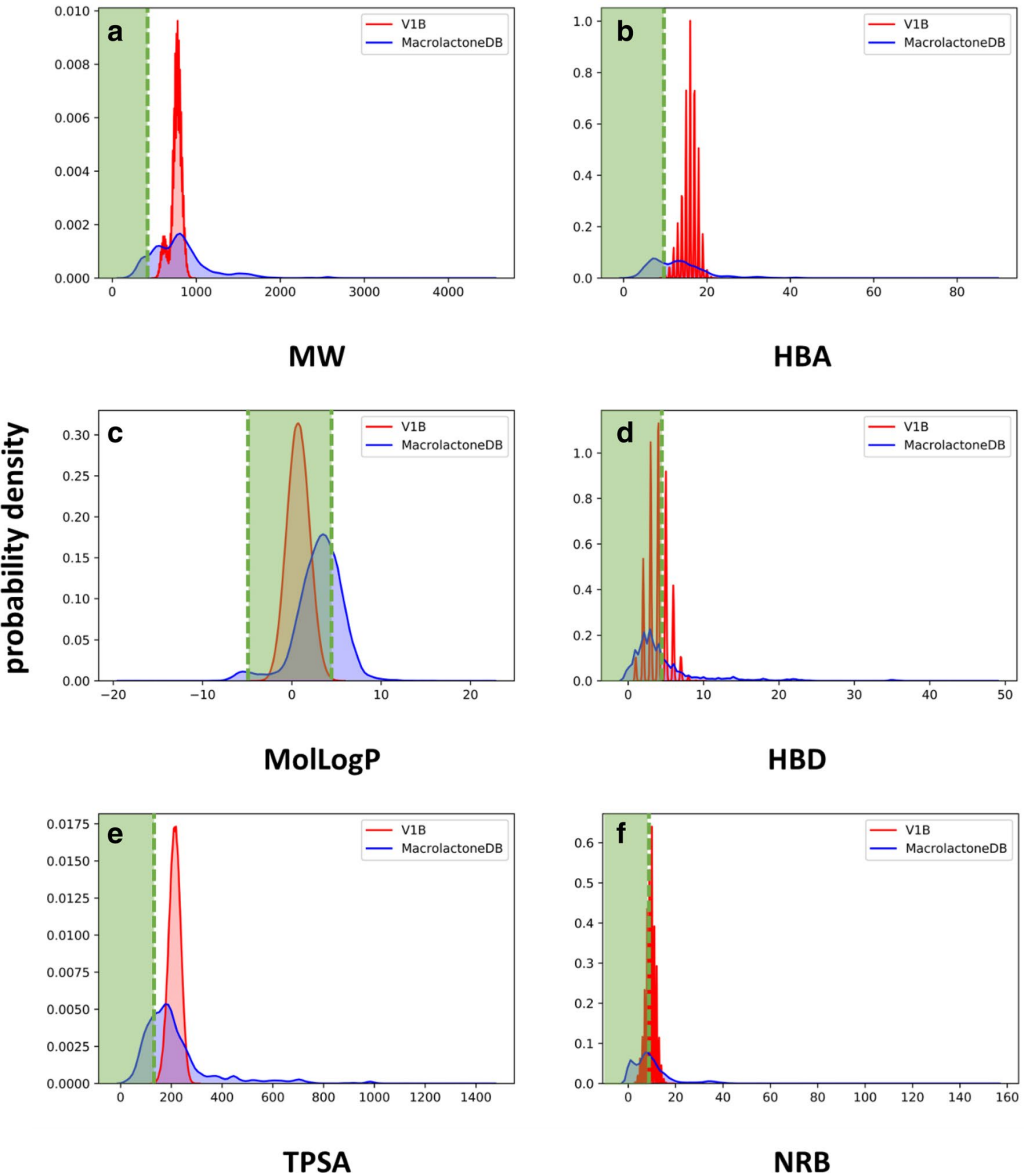
Comparison between density plots of V1B and MacrolactoneDB for molecular properties: **a** MW—molecular weight, **b** MolLogP—calculated water/octanol partition coefficient, **c** TPSA—topological polar surface area, **d** HBA—hydrogen bond acceptors, **e** HBD—hydrogen bond donors, **f** NRB—rotatable bonds. Green rectangles indicate values within druglike regions based on Lipinski and Veber’s rules

To better understand the distribution of macrolide chemical properties in a similar chemical scope, we compared it to the same molecular properties from MacrolactoneDB [26], a recently developed database of ca. 13,700 known macrolactones extracted from public repositories (e.g., ChEMBL, BindingDB, AfroDB, PDB). As it can be seen in the resulting density plots (Fig. 6), V1B macrolides fell within the well-populated regions of MacrolactoneDB across all the molecular properties selected for assessment. This was especially true for MW and TPSA of V1B which were defiantly beyond Lipinski and Veber’s rules. This showed that though V1B might violate one (or several) Lipinski and Veber’s rules commonly used for the assessment of drug likeness and bioavailability of small molecules, V1B macrolides possessed molecular properties within the reasonable ranges displayed by known bioactive macrolactones and macrolides. Of note, none of the key properties studied of V1B were found beyond those displayed by MacrolactoneDB, thus confirming that V1B macrolides were generated structures with acceptable molecular properties being entirely within the chemical space displayed by the currently known macrolactones.

**Fig. 7:**
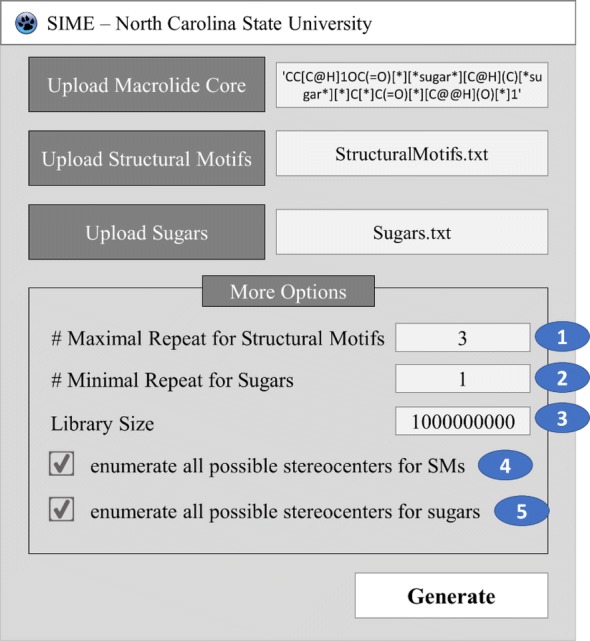
Graphical user Interface of SIME

## Discussion

One major goal of this study was to develop a more synthetic-friendly approach compared to the PKS enumerator from our previous study [16]. Herein, we have addressed this by developing a novel cheminformatics approach that closely follows the way synthetic chemists and biologists could potentially design novel macrolides. SIME can act as a powerful technology to design in silico macrolide libraries with biosynthetic feasibility. This is done by exploiting the knowledge from enzymatic assembly lines that construct macrolides in Nature, and from artificial biosynthetic pathways to diversify macrolides; especially the insights into what extenders can be inserted or manipulated throughout the macrolide scaffolds. These libraries can then be computationally screened via cheminformatics approaches such as QSAR modeling and 3D molecular docking against interesting biological targets to identify lead compounds which can indeed be experimentally tested. Importantly, the insights gained from such experimental results can be used to validate and improve the cheminformatics approaches along with other existing in silico techniques such as prime macrocycle conformational sampling [27], ConfBuster [28], LoopFinder [29]. Additionally, such generated ensembles of macrolides can be further probed and examined for binding profile studies, ligand–protein interactions, conformational arrangements using classic cheminformatics simulations such as molecular dynamic simulations, docking and modeling. This can provide important biological insights into macrolides and expand our knowledge of this structural class and help us design more effective macrolide therapeutics.

It is also important to note that both PKS enumerator [16] and SIME are vital in their own way for investigating the chemical space and features of macrolides. Though SIME can generate VS-ready macrolides that can be more synthetically feasible, PKS enumerator can be used to diversify and generate large ensembles of novel macrolide scaffolds that can be virtually screened against protein targets still. Though these macrolides may be less synthetic-friendly with current biosynthetic technology, binding studies, important protein–ligand interactions and SAR studies can still be extracted to advance hit optimization efforts. The findings from such studies can then be used to guide the direction of synthetic biology towards prioritized novel scaffolds. Thus, PKS enumerator can be useful in expanding the chemical scope of macrolides, discovering their pharmacokinetic features and directing synthetic efforts for certain biological targets. In a future when highly complex enzymatic assembly lines responsible for the production of polyketides can be easily assessed and modified as needed to create novel macrolides, PKS enumerator would be a highly effective and useful technology for efficient screening, identification and SAR studies of macrolactones/macrocycles that can in reality be synthesized and experimentally tested. Meanwhile, the SIME technology can leverage the findings and discoveries from synthetic biology of polyketides to efficiently generate new synthesizable macrolide analogues and help aid in identifying those with high therapeutic potential and extracting SAR knowledge.

We also analyzed the distribution of important chemical properties of 1 million compounds that have been selected with randomly stratified sampling approach from the whole V1B database. Molecular weight, topological polar surface area and hydrogen bond acceptors of V1B macrolides overwhelmingly breached Lipinski’s and Veber’s rules of drug likeness and bioavailability. However, their properties were found to be within the chemical scope of MacrolactoneDB, an *umbrella* structural database of bioactive macrolactones. One may point out that macrolide scaffolds from V1M generated by PKS enumerator in the first study were shown to display a majority of the molecular properties well-within Lipinski’s and Veber’s rules including MW and TPSA and high chemical similarity to experimentally confirmed bioactive molecules [16]. However, in that study, the sugars were not included and only the macrolide scaffolds were considered; both for V1M and 18 reference bioactive macrolides (BMs). The exclusion of sugars significantly impacted the range of chemical properties displayed by both V1M and 18 BMs, thus their properties fell within Lipinski’s and Veber’s druglike and orally bioactive space. If, however, the sugar components were accounted for, the molecular properties such as MW of V1M and 18 BMs would be increased by approx. 352 g mol^−1^ since macrolide antibiotics often contain two sugar moieties such as Cladinose (MW of 176.21 g mol^−1^) and Desosamine (MW of 175.23 g mol^−1^) [30].

Importantly, it should be noted that 3D structures of macrolides are highly important for their chemical properties and biological activities. The 2D molecular descriptors used in this study were generated by RDKit software [31] and used to calculate the physical chemical properties of the enumerated compounds. However, properties such as TPSA, logP or logS can be considerably affected by the 3D conformational arrangements and chameleonic properties usually displayed by macrocyclic structures [32]. For example, we found that experimental logP values of several bioactive macrolides such as azithromycin [33], clarithromycin [33] and erythromycin [33], Carbomycin [33], were at least 1.5 log units higher than the calculated MolLogP values (see Additional file 2: Table S2). However, for many other bioactive macrolides such as oleandomycin and yylosin, the calculated MolLogP values were found to be much more reliable (within 0.8 log unit compared with experimental logP). All the values of calculated MolLogP and experimental logP [33] for these bioactive macrolides are given in Additional file 2: Table S2. Henceforth, the distributions of computed molecular properties such as MolLogP and TPSA for 1 M samples of V1B given in this paper need to be seen as overall trends. Again, this is directly due to the fact that those molecular properties are largely modulated by 3D conformational arrangements of macrolides [32], and the full characterization of the conformation space for the entire library was beyond the scope of this paper.

In fact, the conformational analysis of macrocycles still remains a highly challenging problem and there needs to be further research, method developments and experimental validations in that area. We actually encourage interested readers to consult further studies regarding specific macrocyclic structures and their conformational arrangements such as the conformational control of macrocycles by remote structural modification by Appavoo et al. [34], the prediction of the bioactive conformations of macrocycles using molecular dynamics and docking by Ugur et al. [35], or the conformational exploration study of dissymmetric macrolides antibiotics by Belaidi et al. [36]. SIME generates macrolide structures in SMILES format that can be further processed to generate 3D conformations by using cheminformatics tools (e.g., Prime macrocycle conformational sampling [27], ConfBuster [28]).

Regarding the implementation of the SIME software, we plan to further optimize the algorithm in the future to boost the efficiency in generating libraries and memory storage (e.g., GPU acceleration). We will take into consideration user feedback and incorporate new, better options in our software to help scientists design better in silico macrolide libraries to be used for VS screening, modeling and other cheminformatics studies. We expect to figure out the details on stereochemistry specificity for SMs and sugars at the connecting points to the scaffolds in the near future to better accommodate the findings from biosynthetic chemists. The best potential of SIME in exploring and understanding such complex and enigmatic structural class of macrolides can only be achieved through collaboration between experimental and cheminformatics scientists.

## Conclusions

In this study, we implemented SIME, an efficient chemical library generation software to generate virtual screening-ready macrolide libraries with enhanced biosynthetic feasibility. As proof-of-concept, we utilized SIME to construct V1B, the largest publicly accessible library of 1 billion in silico macrolides based on the core of Erythromycin structure, 13 SMs and six sugars extracted from eighteen bioactive macrolides from the previously conducted study [16]. This new enumeration algorithm relies on the biosynthetic engineering concept of macrolides and insights from such fields to explore and design in silico library of highly biosynthesize-able macrolides. That is highly valuable because the entire library including the lead macrolides predicted by cheminformatics tools such as virtual screening and/or QSAR modeling can in fact be biosynthesized and tested for experimental activities. The highest potential and success of this polyketide enumeration technology in drug discovery can be achieved through collaboration between cheminformatics and biosynthetic studies of macrolides. This can highly impact and contribute to the future studies in search of novel bioactive macrolides.

## Methods

### User controls of SIME

SIME takes three major inputs: (1) macrolide core, (2) structural motifs (SM), and (3) sugars, along with some other user-defined constraints. Users can input the core macrolide structure indicating positions of replacement for sugars and SMs. The macrolide core must be in SMILES format with possible replacement points for SMs designated as asterisks ‘[*]’ and sugars as ‘[*sugar*]’. For example, the erythromycin core with possible places for replacements (see Fig. 1) should be formatted as ‘CC[C@H]1OC(= O)[*][*sugar*][C@H](C)[*sugar*][*]C[*]C(= O)[*][C@@H](O)[*]1’. Structural motifs and sugars can be uploaded as separate.txt files containing the SMILES of SMs and sugars in each line (see software repository for examples of input files). Each SM or sugar should start and end with [*R*] which are connection points to the rest of the core macrolide ring structure or SMs or sugars. One should ensure that between ‘[*R*]’s are the SMILES entailing the desired SM or sugar in SMILES format. The structures of SMs and sugars used to generate V1B are shown in Figs. 2  3 respectively, and the corresponding input text files for SMs and sugars have been provided in the supplementary material of this paper. The structures and IDs of SMs used to generate V1B library were directly extracted from PKS enumerator [16], and the sugar structures were derived from 18 bioactive macrolides from the same cheminformatics study conducted by Zin et al. [16].

**Fig. 8:**
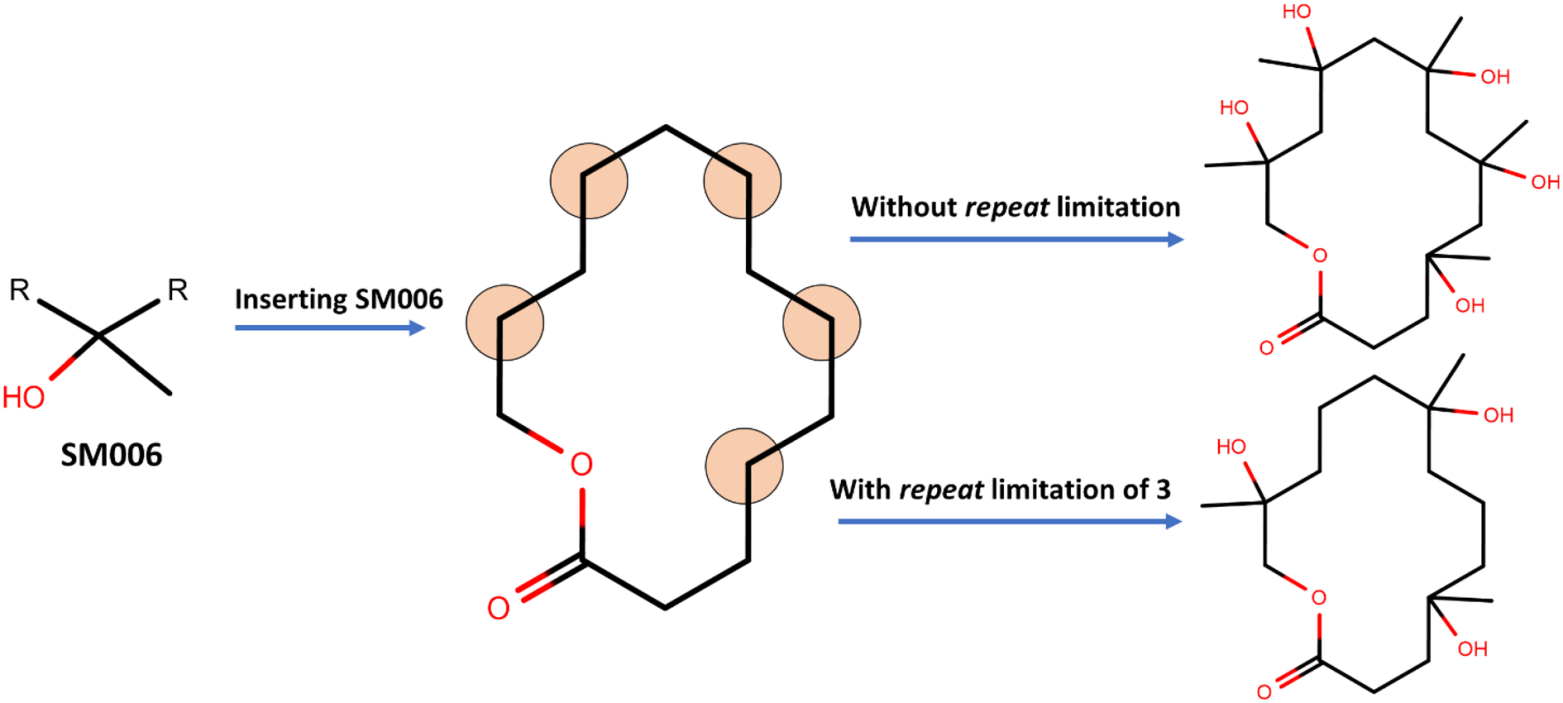
Graphical illustration of the first parameter in SIME; # maximal repeat for SMs

The first parameter in designing the macrolide library was to limit the number of repeated structural motifs in each macrolide (i.e., # maximum number of repeats for SMs). An illustration has been provided in Fig. 8. There might be multiple points of interest for inserting SMs in the macrolide cores and it is likely that macrolides with the same SMs in all these points of interest might be produced. The users can limit the chemical space of macrolide library by choosing the maximum number of SM repeats allowed at those positions. All SMs of interest can then be incorporated into the macrolide cores in the specified positions and may repeat maximally up to that user-defined number per scaffold to enumerate macrolides.

**Fig. 9:**
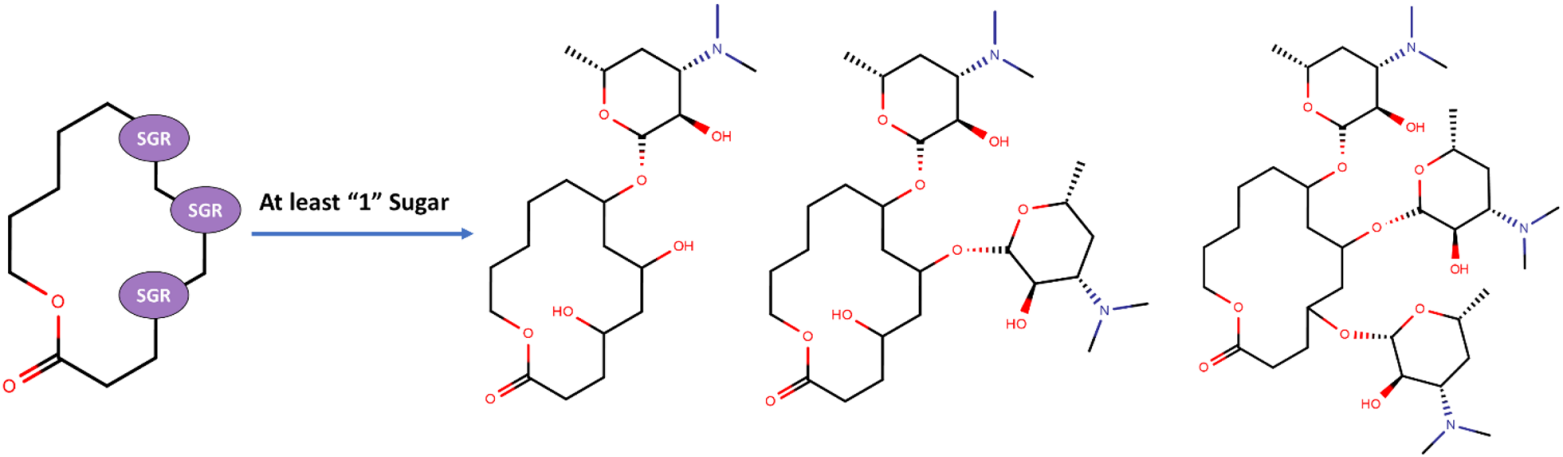
Graphical illustration of the second parameter in SIME; minimal # of sugars

We additionally incorporated a second parameter to control the number of sugars at the specified positions. A graphical illustration has been provided in Fig. 9. A macrolide core may have three (or more) positions at which sugars can be added. However, users may not want macrolides with sugars added in all the positions. This option allows users to specify the minimal number of sugars to add in those positions for the generated macrolides. Users may choose to add at least two sugars to those scaffolds, so the library will contain macrolides with two or three sugars. The rest of the positions which were specified for sugar(s) will have hydroxyl groups attached.

**Fig. 10:**
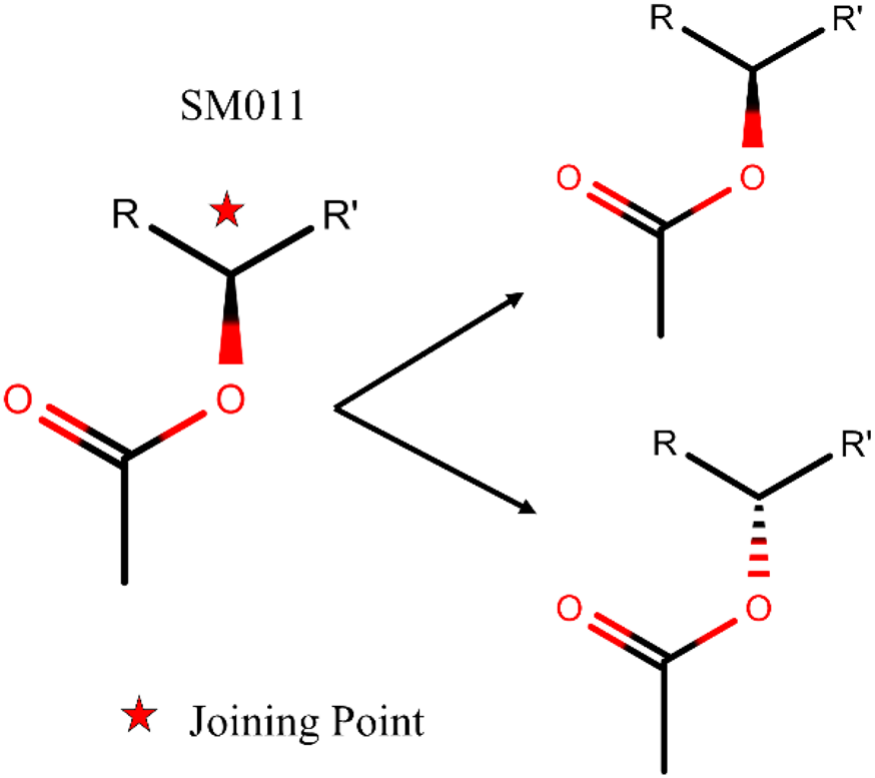
Graphical illustration of the fourth parameter in SIME; enumerate all possible stereocenters. Upon detection of stereocenters at the connecting atom present in SM011, SIME generates both R and S configurations for the joining atom upon user’s request

The third parameter is the library size, the total number of macrolides of the generated library. In the output smile files, each file is limited to at most 1 million. In V1B library, there are in total 1000 files, each containing 1 million macrolides. The fourth and fifth parameters are options to enumerate all possible stereocenters of the connecting atoms for selected SMs and sugars in each scaffold respectively (graphical illustration in Fig. 10). The joining atoms with defined stereocenters in selected SM and sugar are detected by the algorithm and both R and S configurations for those SMs and sugars can be generated at the connecting atoms upon user’s choice. However, joining points with undefined stereocenters are undetected for generation of both R and S configurations. Currently, the backend of SIME is built on RDKit which hasn’t yet obtained the functionality to detect, manipulate or fix R or S configuration at a given stereocenter. As such, the present SIME algorithm does not identify R or S configuration and generates both when a stereocenter is detected at the joining point.

Since very large chemical libraries of macrolides can be generated, output files are sorted in a folder called “LIBRARIES”. The files in the folder are time stamped and named numerically in this format “*timestamp*_mcrl_*. smiles” wherein * stands for the current file number and *timestamp* is replaced with the actual time stamp in the format of “year–month–date–hour–minute–second”. Additionally, the program outputs an information file entailing all the user parameters applied to generate the program along with the macrolide core, sugars and structural motifs that users have selected.

### Implementation details

**Fig. 11:**
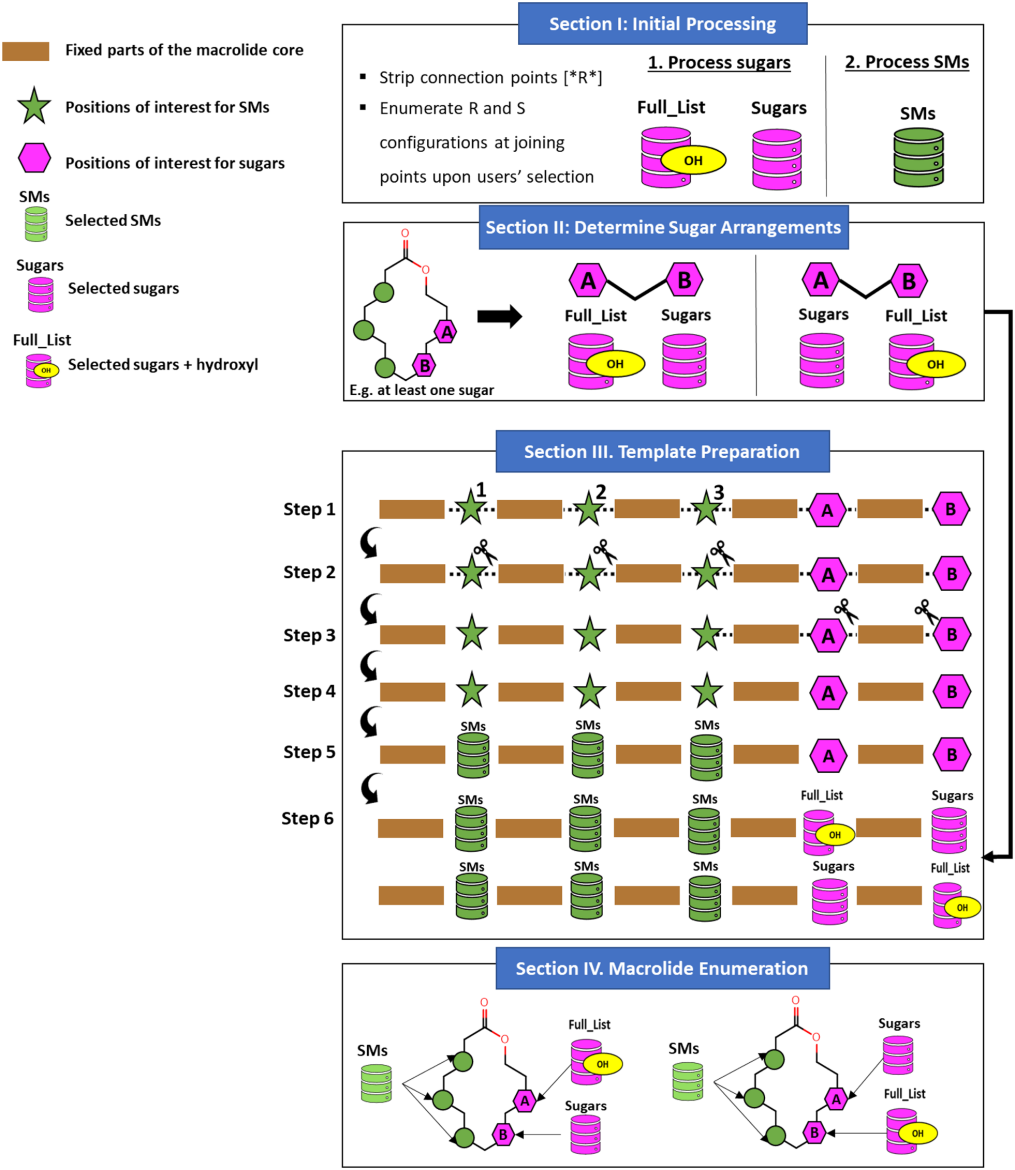
Graphical illustrative workflow of the core SIME. Sections I, II, III and IV are explained in the implementation details of “Methods” section

The inputs for SIME are (1) macrolide core, (2) SMs, (3) sugars, (4) parameters 1-4 (Fig. 2) and the required formats for the first three inputs are provided in User Controls of SIME. The output is a folder containing *smiles* files each of which contains a maximal of 1 million macrolide structures encoded in chemical SMILES format. It can be divided into three major sections: (I) initial processing, (II) template preparation and (III) enumeration and creation of macrolides. The graphical illustration for the simplified workflow of SIME is provided in Fig. 11.

In the first section of initial processing, sugars are processed. The program loads sugars from a selected file (currently set to default sugar file). If users choose not to enumerate both R and S configurations of sugars at joining points (parameter 5th), connection points [*R*] for each sugar are stripped and then appended to ‘*Sugars’* list. If users select 5th parameter, the function ENUMERATE_sugar_stereocenters (**F**_**SG_RS**_, Table 1) is performed for each sugar to generate both R and S configurations of sugars at joining points. These sugars are then appended to *sugar* list. Another list ‘*Full_List*’ is created by adding a hydroxyl to ‘*Sugars’* list. In step 2, SMs are processed. The file containing SMs of users’ choice is loaded into the program (currently from default SM file). If users choose not to enumerate both R and S configurations of SMs at joining points (4th parameter), connection points [*R*] for each SM are stripped and then appended to *structural_motifs* list. If users select the 4th parameter, the function enumerate_SM_stereocenters (**F**_**SM_RS**_, Table 1) is performed for each SM to generate both R and S configurations at joining points and then appended to *structural_motifs* list.

**Table 1:** Helper functions for SIME. The descriptions for each function were provided to help understand the simplified workflow of SIME algorithm provided in Fig. 11

Helper functions	Description
ENUMERATE_sugar_stereocenters (**F**_**SG_RS**_)	Take in sugar strings that start and end with [*R*] and return a list of sugars with two different stereocenters for the joining carbon
enumerate_SM_stereocenters (**F**_**SM_RS**_)	Takes a list of SMs. For SMs with identified stereocenters at the joining point, both R and S configurations for those SMs are generated and added to the *all_possible* list. For SMs with undefined stereocenters at joining points or without stereocenters, they will remain unchanged and added to the *all_possible* list. Returns the *all_possible* list
remove_SM_digits (**F**_**RSMD**_)	Takes a given smile and locates SM points of interest indicated with [1*], [2*], etc. Returns the smile string with all SM points of interest with removed digits Input — > ’1[1*]234[2*]5[3*]6’ Output — > ’1[*]234[*]5[*]6’
string_splitter (**F**_**SS**_)	Splits a given string into fragments based on a symbol provided and returns a list containing the fragments. For example: input — > string = ’1[*]234[*]5[*]6’, symbol = ’[*]’ output — > [‘1’, ‘[*]’, ‘234’, ‘[*]’, ‘5’, ‘[*]’, ‘6’]
insert_SMs (**F**_**iSM**_)	Takes in a smile template resulted from string_splitter and replace the ‘[*]’ symbols with a list of SMs
generate_dummy_sugar_templates (**F**_**GDST**_)	This function takes two parameters: smile template, minimal sugars in each macrolide (default is one sugar). For simplification purposes, it generates a list of all possible sugar dummies as ‘*SUGARS*’ (intended for only sugars) and ‘*Full_List*’ (intended for sugars + hydroxy) for the number of sugars specified in the given smile template. For example, if there are two sugar positions identified in the given core with at least one sugar allowed, this function will output this result: [(‘*SUGARS*’, ‘*Full_List*’), (‘*Full_List*’, ‘*SUGARS*’)]. It means that the first and second locations for sugars in one template allow for the list of ‘*SUGARS*’ and ‘*Full_List*’ (sugar + hydroxyl) respectively. The second template allow for the full list and the list of sugars in the first and second locations designated for sugars respectively
replace_SYMBOLsugars_with_dummies (**F**_**RSSD**_)	This function takes two inputs: sugar_dummy_order and smile_template_with_sugar_symbols. It splits the given template at [*sugar*] positions wherein the correct dummies (‘SUGARS’ and ‘Full_List’) are inserted
insert_sugars_to_dummies (**F**_**IStD**_)	This function takes the smile template with specified ‘SUGARS’ and ‘Full_List’ after *** function. It then replaces ‘SUGARS’ with an actual list of sugars, and ‘Full_List’ with the list of sugars and a hydroxyl group

In section II, the 2nd parameter, minimal repeat for sugars, is addressed. Wherein sugars are not used at sugar positions of interest in the macrolide core, a hydroxyl functional group is used in its place. Thus, two lists of ‘*Sugars*’ and ‘*Full_List’* were made in the first section of initial processing. ‘*Full_List’* contains ‘*Sugars*’ and a hydroxyl group. The total possible arrangements of ‘*Sugars*’ or ‘*Full_List*’, according the 2nd parameter (Fig. 7), to be inserted at sugar positions of interest in the macrolide core template are determined using the function generate_dummy_sugar_templates (**F**_**GDST**_, Table 1).

In the third section of template preparation, the core macrolide is processed and prepped for the creation of new macrolide structures. The graphical illustration for the simplified workflow for this section is provided in Fig. 11. Of note, the default for core macrolide is set to erythromycin core provided in Fig. 1 and as of now, only the first single macrolide core provided in the text file is considered. In step 1, the function remove_SM_digits (**F**_**RSMD**_, Table 1) is applied to the core macrolide smile string. It strips the labeled digits from the SM positions of interest if there are any. For example, **F**_**RSMD**_ converts ‘–[1*]–[2*]–[3*]—[*sugar*]–[*sugar*]’ into ‘–[*]–[*]–[*]—[*sugar*]–[*sugar*]’. In step 2, string_splitter (**F**_**SS**_) is applied to the output smile from step 1 to split into different fragments. There may be fragments in which sugar positions of interest may be embedded. Thus, in step 3, all the fragments are assessed for the presence of sugar positions of interest and again split at those sugar positions into more fragments. The fragments are then cleaned up for further processing. In step 4, *structural_motifs* from section I are inserted at SM positions of interest in the macrolide core template. In step 5, based on the arrangements of sugar lists calculated in section II, ‘*Sugars*’ and ‘*Full_List*’ are inserted at sugar positions of interest in the macrolide core template. Now, the lists of SMs and sugars are prepped and placed at the right positions of the macrolide core template, thus it is ready to proceed into the section IV.

During section IV, macrolides are enumerated and created in SMILES format based on the prepped macrolide core template from section III. The cartesian products (CP) of all the fixed fragments, ‘*structural_motifs*’, ‘*Sugars*’ and ‘*Full_List*’ at the specified positions are generated to enumerate possible macrolide structures. During this enumeration process, the program checks if the total number of repeatable SMs in the macrolide (1st parameter in Fig. 7) is satisfied after the enumeration of a macrolide. If so, the program proceeds to check whether the library size has been achieved. If not, the next cartesian product is enumerated and a new macrolide structure is created. This process continues until the desire library size is achieved or the internal memory capacity is full.

The full code of SIME (and the link to download the full V1B library) is freely available for download at https://github.com/zinph/SIME

## Supplementary information

**Additional file 1.** List of 1 million SMILES strings extracted from the V1B library.

**Additional file 2.** Table S1: Descriptive Statistics on the Molecular Properties of V1B. Table S2: Calculated MolLogP vs. experimental LogP of some known bioactive macrolides.

## Abbreviations

SIME: Synthetic insight-based macrolide enumerator
PKS: Polyketides
SM: Structural motifs
V1B: Virtual 1 billion macrolides (with sugars incorporated into macrolide scaffolds)
VS: Virtual screening
MW: Molecular weight
SlogP: Hydrophobicity
TPSA: Topological polar surface area
HBA: Hydrogen bond acceptors
HBD: Hydrogen bond donors
NRB: Number of rotatable bonds
