# Supplementary material for: SIME: synthetic insight-based macrolide enumerator to generate the V1B library of 1 billion macrolides
Source: J Cheminform. 2020 Apr 10;12:23. doi: 10.1186/s13321-020-00427-6 (PMC7146965; doi:10.1186/s13321-020-00427-6)
Supplement: Supplementary file 2 — Additional file 2. Table S1: Descriptive Statistics on the Molecular Properties of V1B. Table S2: Calculated MolLogP vs. experimental LogP of some known bioactive macrolides. [file 13321_2020_427_MOESM2_ESM.docx]

**Additional file 2**

SIME & V1B: 1 Billion Macrolides for Virtual Screening

*Phyo Phyo Kyaw Zin^1,2^, Gavin Williams^1,3^, and Denis Fourches^1,2,3^**

^1^ Department of Chemistry, North Carolina State University, Raleigh, NC, USA.

^2^ Bioinformatics Research Center, North Carolina State University, Raleigh, NC, USA.

^3^ Comparative Medicine Institute, North Carolina State University, Raleigh, NC, USA.

* To whom correspondence should be sent. Email: [dfourch@ncsu.edu](mailto:dfourch@ncsu.edu)

**Table S1.** Descriptive Statistics on the Molecular Properties of V1B.

|  | *MW* | *TPSA* | *HBA* | *HBD* | *NRB* | *MolLogP* |
| --- | --- | --- | --- | --- | --- | --- |
| Mean | 761.25 | 213.09 | 15.91 | 3.94 | 9.50 | 0.73 |
| Standard Error | 0.07 | 0.02 | 0.00 | 0.00 | 0.00 | 0.00 |
| Median | 770.86 | 213.59 | 16.00 | 4.00 | 10.00 | 0.72 |
| Mode | 791.93 | 209.21 | 16.00 | 4.00 | 10.00 | 1.32 |
| Standard Deviation | 68.17 | 22.88 | 1.77 | 1.35 | 2.00 | 1.24 |
| Sample Variance | 4647.79 | 523.30 | 3.15 | 1.83 | 4.02 | 1.53 |
| Range | 486.47 | 181.11 | 13.00 | 9.00 | 16.00 | 9.96 |
| Minimum | 488.62 | 125.76 | 9.00 | 1.00 | 3.00 | -4.30 |
| Maximum | 975.09 | 306.87 | 22.00 | 10.00 | 19.00 | 5.66 |
| Sum | 7.61E+08 | 2.13E+08 | 1.59E+07 | 3.94E+06 | 9.50E+06 | 7.29E+05 |
| Count | 1.00E+06 | 1.00E+06 | 1.00E+06 | 1.00E+06 | 1.00E+06 | 1.00E+06 |
| Confidence Level (95.0%) | 0.13 | 0.04 | 0.00 | 0.00 | 0.00 | 0.00 |

**Table S2.** Calculated vs. experimental LogP for known bioactive macrolides.

| Macrolides | Cal. MolLogP | Exp. LogP |
| --- | --- | --- |
| Azithromycin | 0.525 | 4.02 |
| Clarithromycin | 1.064 | 3.16 |
| Erythromycin | 0.41 | 3.06 |
| Oleandomycin | 0.923 | 1.69 |
| Tylosin | 1.981 | 1.63 |
| Carbomycin | 1.144 | 3.04 |
